# Expanding the neurological and behavioral phenotype of White-Sutton syndrome: a case report

**DOI:** 10.1186/s13052-021-01101-9

**Published:** 2021-07-02

**Authors:** Bernadette Donnarumma, Maria Pia Riccio, Gaetano Terrone, Melania Palma, Pietro Strisciuglio, Iris Scala

**Affiliations:** 1 Department of Translational Science, Federico II University Hospital, Via S. Pansini 5, 80131 Naples, Italy; 2 Department of Maternal and Child Health, Federico II University Hospital, Via S. Pansini 5, 80131 Naples, Italy

**Keywords:** White-Sutton syndrome, POGZ, POGZ mutation, Epilepsy, Paroxysmal not-epileptic events, EEG abnormalities, Cognitive profile, Autism, Case report

## Abstract

**Background:**

White-Sutton (WHSUS) is a recently recognized syndrome caused by mutations of the POGZ gene. Approximately 70 patients have been reported to date. Intellectual disability, hypotonia, behavioral abnormalities, autism, and typical facial dysmorphisms are recognized as WHSUS features; however, still few patients receive a comprehensive psychometric, behavioral and neurological examination. In this report, we describe the pediatric, dysmorphological, neurological, psychometric and behavioral phenotype in a new WHSUS patient due to a novel heterozygous POGZ mutation, highlighting the distinctive epileptic phenotype and the cognitive pattern.

**Case presentation:**

The patient, an 8 years-old girl, presented history of hypotonia, motor and speech delay, and distinctive facial features. The diagnosis of WHSUS followed the identification of the de novo variant p.Asp828GlyfsTer36 (c.2482dupG) in the POGZ gene. The patient showed a distinctive neurological phenotype with the occurrence of both paroxysmal not-epileptic events in the first 6 months of age and EEG abnormalities without evidence of clinical seizures after the first year of age. Psychological and behavioral testing highlighted moderate intellectual and communication deficit, mild autism spectrum and visual-motor integration deficit.

**Conclusions:**

This is the first described case of WHSUS with a co-existence of paroxysmal not-epileptic events and abnormal EEG without seizures in the same patient. Together with the available literature data, this observation suggests that paroxysmal not-epileptic events could be more frequent than expected and that this feature belongs to the WHSUS phenotypic spectrum. Autism is a known comorbidity of WHSUS but is still poorly investigated. Specific clinical testing could help detect also mild autistic phenotypes and better define autism prevalence in POGZ-related syndrome. Special attention should be given to symptoms such as stereotypies, social withdrawal, and hyperactivity that, when present, should be considered as possible signs of autism symptoms. The dissection of the neurological and behavioral phenotype is crucial for individualized therapies tailored to patient’s needs.

## Background

White-Sutton (WHSUS, MIM #616364) is a recently recognized syndrome caused by mutations of the POGZ (pogo transposable element-derived protein with zinc finger domain) gene, encoding for a multidomain nuclear protein that regulates chromatin remodeling, chromosome segregation and mitotic progression, making the majority of cells to prematurely exit mitosis with consequent depletion of neurogenic progenitor cells [1, 2].

WHSUS is characterized by developmental delay, intellectual disability, hypotonia, behavioral abnormalities, autism, and typical facial dysmorphisms [3]. Patients may also present short stature, microcephaly, cerebral malformations, strabismus, visual impairment, hearing loss and gastrointestinal disorders such as feeding difficulties, gastroesophageal reflux, and constipation [4]. The definition of the specific neuropsychological pattern of WHSUS is still ongoing. A recent retrospective study of 19 subjects affected by WHSUS outlined differences in intellectual efficiency, ranging from learning disability to severe intellectual deficit, and the presence of behavioral disorders including anxiety, stereotypies, social withdrawal, and hyperactivity. Language, attention, executive and social skills were mostly impaired in the cohort [5]. Researchers underline that only a minority of patients with WHSUS undergo to specific psychometric testing able to distinguish between intellectual and learning disability and that few of them perform behavioral tests [5]. Indeed, in this first neuropsychological evaluation of a WHSUS cohort, only one patient was specifically tested for autism.

The epileptic phenotype in WHSUS is an additional clinical feature deserving further investigation. To the best of our knowledge, only 14 patients with WHSUS and epilepsy were reported in the literature. Medical reports plus EEG were available in 7 patients [4, 6–8]; among these, paroxysmal not-epileptic events were described in 1 subject [7] while EEG abnormalities without clinical seizures in 2 subjects [6] (Table 1). The co-existence of both paroxysmal not-epileptic events and abnormal EEG without clinical seizures in the same subject was never described so far.

**Table 1:** Genetic and phenotypic characterization of the present case and comparison with other WHSUS patients with paroxysmal not-epileptic events or EEG abnormalities without seizures

	Present case	Ferretti et al. 2019 [7]	Stessman et al. 2016 [6]	
Gene variant (POGZ gene: NM_015100.3)	p.Asp828GlyfsTer36 (c.2482dupG)	p.Leu904* (c.2711 T > G)	p.Gln1283* (c.3847C > T)	p.Glu1154Thrfs*4 (c.3456_3457del)
Age at onset	3 months	4 months	n.r.	n.r.
Age at diagnosis	8 years	n.r.	n.r.	n.r.
Dysmorphic features	High and broad forehead; Bitemporal narrowing; Hypertelorism; Up-slanting and long palpebral fissures; Midface hypoplasia; Broad nasal bridge, anteverted nares; Protruding tongue; Macrostomy; Incisors diastasis; Clinodactyly of the fifth fingers; 4th toes brachydactyly and clinodactyly; Sandal gap.	High and broad forehead; Bitemporal narrowing; Epicanthus; Broad nasal bridge; Macrostomy; Down-turned corners of the mouth; Poinetd chin; Clinodactyly.	Brachycephaly; High nasal bridge, slight deviation of the nose, upturned tip of the nose; Thin upper lip.	Brachycephaly; Flat midface; Hypertelorism; Epicanthic folds.
Growth	Height 25th centile, BMI z-score 1.84	n.r.	Height 0.6th centile, BMI z-score 1.8	Height 30th centile, BMI z-score 1.8
Microcephaly	–	+	+	–
Motor skills	Hypotonia; Motor delay; Fine motor skill deficit; Visual-motor integration deficit; Clumsiness.	Hypotonia; Severe psycho-motor delay.	Mild/moderate motor delay	Mild motor delay
Language	Speech delay; Echolalia	Absent speech	Language skills regression	Speech delay
Intellectual disability	IQ 60	Severe	Severe	IQ of 55 (at 6 y)
Autism	+	+	–	–
ADHD	–	n.r.	n.r.	n.r.
Seizures	Paroxysmal not-epileptic events; EEG abnormalities without epilepsy (sharp waves over the biemispheric centro-temporal areas)	Paroxysmal not-epileptic events; EEG abnormalities with epilepsy (bitemporal frontal spike-and-waves abnormalities)	EEG epileptic abnormalities without epilepsy (bilateral frontal abnormalities)	EEG epileptic abnormalities without epilepsy
Brain imaging	Lateral ventricle enlargement; Fronto-temporo-parietal cortical atrophy.	Cortical and subcortical cerebral atrophy associated with enlargement of the third ventricle and temporal horns of lateral ventricles; thin corpus callosum	n.r.	n.r.
Eye abnormality	Esotropia	–	–	High hypermetropia
Hearing loss	–	+ (sensorineural)	n.r.	+ (conductive)
Gastrointestinal involvement	–	Poor feeding; Gastric distension.	Feeding problems	
Sleep disorders	–	n.r.	+	n.r.
Congenital Heart Defect	–	n.r.	n.r.	n.r.
Other	Dorsal Hyperkyphosis	Visual inattention	anxiety, self mutilation	n.r.

*ADHD* Attention deficit/hyperactivity disorder, *IQ* Intelligent quotient, +: present – : absent, *n.r.* data not specifically reported in the cited studies

Here we report the pediatric, dysmorphological, neurological, psychometric and behavioral phenotype of a new WHSUS patient due to a novel heterozygous POGZ mutation, highlighting the distinctive epileptic phenotype, characterized by both paroxysmal not-epileptic events and EEG abnormalities without epileptic seizures, and the cognitive pattern.

## Case presentation

**Fig. 1:**
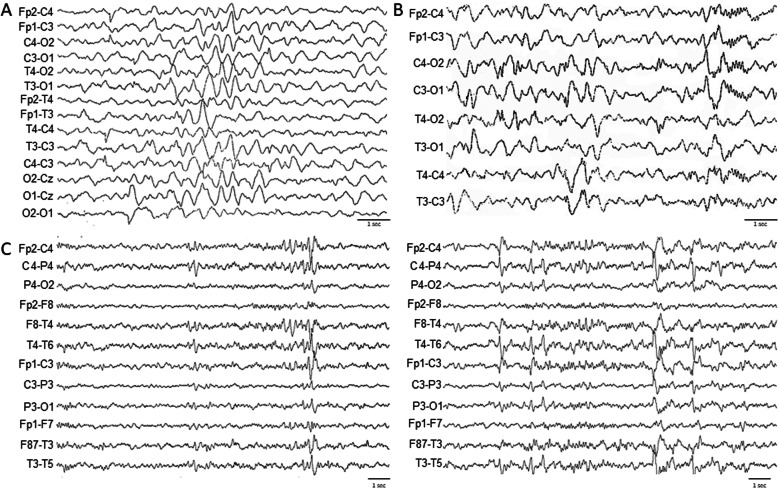
Electroencephalograms at 6 months (**A**), 14 months (**B**) and 8 years old (**C**)

The patient was the second child of two Caucasian non consanguineous parents. She was born by caesarean section and the perinatal history was uneventful. Her sister was affected by selective mutism. Since the first months of life, the patient presented hypotonia with consequent delay of the developmental milestones as she could sit without support at 12 months and move the first independent steps at 22 months. The child pronounced her first words at 13 months, but never achieved appropriate language skills. Bowel and bladder continence was gained at 4 years old. At the age of 3 and 6 months, the patient experienced two paroxysmal not-epileptic events characterized by lower and upper limbs stiffness, reduced response to external stimuli and eye deviation, lasting few seconds. A first sleep electroencephalogram (EEG) at this time (6 months) showed a slow background cerebral activity without epileptic abnormalities (Fig. 1A). An EEG performed at the age of 14 months showed rare spike and wave complexes over both occipital areas, with prevalence in the right hemisphere (Fig. 1B). A brain magnetic resonance (MRI) performed at the age of 3 years old showed an enlargement of the lateral ventricles and of the fronto-temporo-parietal cerebrospinal fluid spaces, associated with a cortical atrophy. At our first evaluation at 8 years old, the girl attended the first year of primary school with a special education program and practiced behavioral therapy. Dysmorphic features were present, such as high and broad forehead, bitemporal narrowing, hypertelorism, up-slanting and long palpebral fissures, midface hypoplasia, broad nasal bridge, anteverted nares, protruding tongue, macrostomy, incisors diastasis, fifth fingers clinodactyly, 4th toes brachydactyly and clinodactyly, sandal gap. She also presented esotropia and dorsal hyperkyphosis. Hearing function was unaffected. No cardiac or gastrointestinal disorders were detected. The BMI z-score was 1.84. Height and head circumference were normal. Chromosomal microarray analysis showed no abnormal results. A multi-gene analysis for neuro-developmental disorders performed using next generation sequencing showed the de novo variant p.Asp828GlyfsTer36 (c.2482dupG) in the POGZ gene (NM_015100.3), not described in the literature so far. The mutation, not reported in the population database (ExAC, EVS, 1000 genome project), causes a frameshift and a premature stop codon at the amino acid 864 over a protein length of 1410 amino acids and is considered as potentially pathogenetic according to the recommendations of the American College of Medical Genetics and Genomics (ACMG) [9]. The patient received a deep neurological and psychiatric evaluation to better define the electroencephalographic, neuropsychological and behavioral phenotypes. Clinical seizures were absent in recent medical history. Sleep EEG showed sharp waves over the bi-emispheric centro-temporal areas (Fig. 1C). At the neurological examination, muscular tone, deep tendon reflexes, cranial nerves and cerebellar function were preserved. A clumsy gait was observed. The patient presented echolalia, stereotypic hand movements, poorly modulated eye contact, repetitive behavior and restricted social interaction. Motor skills were deficient in fine movements. The following questionnaires were administered to parents to assess the presence of behavioral disturbance and psychopathological risk, and resulted in normal scores: the Aberrant Behavior Checklist (ABC) [10], exploring items related to Irritability/agitation/crying, Letargy/social withdrawal, Stereotypic behavior, Hyperactivity/non-compliance and Inappropriate speech; the Conner’s Parent Rating Scales (CPRS) [11], exploring Attention Deficit/Hyperactivity Disorder; and the Child Behavior Checklist for age 6–18 (CBCL) [12].

The Childhood Autism Rating Scale - second edition standard version (CARS-2-ST) [13] showed a total-raw score of 28.5, diagnostic for minimal symptoms of Autism Spectrum Disorders; major difficulties were found in the items “Relating people”, “Object use”, and “Taste, smell and touch response and use”. The Social Responsiveness Scales (SRS) [14] showed a pathological total T-score, with severe impairment of “Social awareness”, “Social motivation” and “Mannerisms” areas. Developmental test of Visual Motor Integration (VMI) showed deficient visual-motor integration (VMI T-score = 69, 2th percentile). Adaptive level, explored by means of the Vineland-II questionnaire [15] showed a Deviation Quotient of 81 (10th percentile), with a specific fall in “Communication Area”. This result was in line with the intellectual evaluation by Leiter-R test [16], which revealed an Intelligence Quotient (IQ) of 60, with a fluid reasoning quotient of 50, suggestive of moderate cognitive impairment. The questionnaires Sleep Disturbance Scale for Children (SDSC) and Behavioral Paediatrics Feeding Assessment Scale (BPFAS) excluded sleep and eating disorders (Table 1).

## Discussion and conclusions

Mutations in the POGZ gene were identified as a novel genetic cause of developmental disorders in 2015 [17, 18]. Since then, approximately 70 individuals have been reported in the literature having various degree of intellectual disability and distinguishing facial features. However, data regarding the full phenotypic spectrum, prognosis and genotype-phenotype correlations are still incomplete.

The present case further defines the mutational and clinical spectrum of POGZ-related syndrome. Besides the association of typical facial features, hypotonia, cognitive impairment and speech delay, some elements of her clinical history should be underlined. In her early life, the patient experienced two paroxysmal not-epileptic events with normal EEG and a later onset of EEG abnormalities in the absence of clinical seizures. To date, the co-existence in the same patient of paroxysmal not-epileptic events and abnormal EEG without seizures has never been reported in the literature. Paroxysmal not-epileptic events were reported in one Italian patient [7]: the child, at 4 months of age, presented paroxysmal episodes characterized by hyperextension of the upper limbs, psychomotor arrest with eye deviation and normal EEG, followed by onset of epileptic seizures requiring therapy at 2 years of age. The authors speculated that paroxysmal not-epileptic events could be an uncommon finding in WHSUS [8]. The present case shares with this patient the age of onset in the first months of life of paroxysmal not-epileptic event and the neurological symptoms characterized by lower and upper limbs stiffness, reduced response to external stimuli and eye deviation. Differently from the previous case, our patient did not develop epilepsy. However, electroencephalographic anomalies were registered from 14-months of age. Abnormal EEG without seizures have been described in two other WHSUS subjects [6] (Table 1). These observations suggest that the dissociation between convulsive episodes and EEG abnormalities could be more prevalent than expected among WHSUS patients and that this feature belongs to the wide WHSUS phenotypic spectrum. A correct diagnosis of paroxysmal not-epileptic events is crucial to avoid unnecessary pharmacological intervention and to define neurological prognosis. The comparison between the present case and other WHSUS patients with paroxysmal not-epileptic events or EEG abnormalities without seizures did not reveal a clear genotype-phenotype correlation. The mutations, inducing premature stop codons, were located in the zinc finger-domain (present case), the prolin-rich domain [7] and the DDE transposase domain [6]. The present case shares with the one described by Ferretti and colleagues [7] the occurrence of paroxysmal not-epileptic events and the presence of cortical atrophy; however, the child here described presented a milder neurological phenotype and no microcephaly. Both patients received a diagnosis of autism. The patients reported by Stessman and colleagues with EEG abnormalities without clinical epilepsy [6] presented a heterogeneous neurological involvement, one being affected by language skill regression, severe behavioral problems and microchephaly, the other by less impaired language skills and normal head circumference. No brain MRI details were provided. Both patients were not affected by autism.

Compared with literature data, this case confirms the presence of a behavioral profile characterized by internalizing problems, intellectual and communication deficit [4, 5]. In addition, the behavioral evaluation highlighted the occurrence of mild autism spectrum. Of note, this condition was diagnosed by means of a neuropsychiatric clinical evaluation and specific diagnostic tests (the Childhood Autism Rating Scale and the Social Responsiveness Scales), while generic questionnaires to parents, assessing behavioral disturbance, including social withdrawal, stereotypic behaviour and communication problems, did not reveal elements of concern. This observation suggests that patients with WHSUS deserve specific evaluation for autism and that questionnaires are helpful instruments for guiding clinicians in the clinical diagnosis of autism. Autism is a known comorbidity of WHSUS but it is still poorly investigated. Specific evaluations could help detect also mild autistic phenotypes and to better define autism prevalence in POGZ-related syndrome. A complete neuropsychological evaluation should be proposed not only to precociously detect subtle autism symptoms, but also to correctly diagnose other possible behavioral disorders.

Finally, patients with WHSUS present impairment of fine motor skills. Our data suggest that fine motor skills deficit may be a consequence of deficient visual-motor integration rather than of general dyspraxia.

In conclusion, the dissociation between convulsive episodes and EEG anomalies suggests the importance of a periodic neurological evaluation and EEG monitoring in all patients affected by WHSUS. All patients should also receive a neuropsychological evaluation and a specific testing to precociously detect Autism Spectrum Disorders for individualized therapies tailored to patient’s needs. Special attention should be given to symptoms such as stereotypies, social withdrawal, and hyperactivity that, when present, should be considered as possible signs of autism symptoms rather than not-specific behavioral disorders.

## Data Availability

Data sharing is not applicable to this article as no datasets were generated during the current study. All data analysed during this study are included in this published article. Clinical data are available in the patient’s clinical records at the Department of Maternal and Child Health, Federico II University Hospital.

## Abbreviations

WHSUS: White-Sutton syndrome
POGZ: Pogo transposable element-derived protein with zinc finger domain
EEG: Electroencephalogram
ACMG: American College of Medical Genetics and Genomics
ABC: Aberrant Behaviour Checklist
CPRS: Conner’s Parent Rating Scales
CBCL: Child Behaviour Checklist for age 6–18
CARS-2-ST: Childhood Autism Rating Scale - second edition standard version
SRS: Social Responsiveness Scales
VMI: Developmental test of Visual Motor Integration
SDSC: Sleep Disturbance Scale for Children
BPFAS: Behavioural Paediatrics Feeding Assessment Scale
ADHD: Attention deficit/hyperactivity disorder
IQ: Intelligent quotient
